# Physiological and Psychological Effects of Forest and Urban Sounds Using High-Resolution Sound Sources

**DOI:** 10.3390/ijerph16152649

**Published:** 2019-07-24

**Authors:** Hyunju Jo, Chorong Song, Harumi Ikei, Seiya Enomoto, Hiromitsu Kobayashi, Yoshifumi Miyazaki

**Affiliations:** 1 Center for Environment, Health and Field Sciences, Chiba University, 6-2-1 Kashiwa-no-ha, Kashiwa, Chiba 277-0882, Japan; 2 Department of Wood Engineering, Forestry and Forest Products Research Institute, 1 Matsunosato, Tsukuba, Ibaraki 305-8687, Japan; 3 JVCKENWOOD Victor Entertainment Corporation, 1-2-20 Higashi, Shibuya-ku, Tokyo 150-0011, Japan; 4 Department of Nursing, Ishikawa Prefectural Nursing University, 1-1 Gakuendai, Kahoku, Ishikawa 929-1210, Japan

**Keywords:** forest sound, natural sound, physiological relaxation effects, prefrontal cortex activity, autonomic nervous activity, near-infrared spectroscopy, heart rate variability, heart rate, semantic differential method, profile of mood states

## Abstract

Exposure to natural sounds is known to induce feelings of relaxation; however, only few studies have provided scientific evidence on its physiological effects. This study examined prefrontal cortex and autonomic nervous activities in response to forest sound. A total of 29 female university students (mean age 22.3 ± 2.1 years) were exposed to high-resolution sounds of a forest or city for 60 s, using headphones. Oxyhemoglobin (oxy-Hb) concentrations in the prefrontal cortex were determined by near-infrared spectroscopy. Heart rate, the high-frequency component of heart rate variability (which reflects parasympathetic nervous activity), and the ratio of low-frequency to high-frequency (LF/HF) components (which reflects sympathetic nervous activity) were measured. Subjective evaluation was performed using the modified semantic differential method and profiles of mood states. Exposure to the forest sound resulted in the following significant differences compared with exposure to city sound: decreased oxy-Hb concentrations in the right prefrontal cortex; decreased ln(LF/HF); decreased heart rate; improved feelings described as “comfortable,’’ “relaxed,” and “natural”; and improved mood states. The findings of this study demonstrated that forest-derived auditory stimulation induced physiological and psychological relaxation effects.

## 1. Introduction

Human physiological functions have adapted to the natural environment over 6–7 million years of human evolution [1,2]. However, since the industrial revolution, an increasing number of people have moved from natural to artificial urban environments. According to a United Nations report, 30% of the world’s population in 1950 was urban population, and this value is projected to rise to 66% by 2050 [3]. In evolutionary terms, human physiology, which has evolved in response to the natural environment, has had little time to adapt to the artificial environments of urban areas. Some researchers have proposed this to be a reason why many people in urban areas experience stress and tension [2,4,5].

In recent years, the relaxing and restorative effects of the natural environment have gradually gained attention [4,5,6,7], with the development of physiological measurement technology facilitating the accumulation of scientific evidence based on physiological parameters. 

Field experiment studies, in which all five senses of participants were involved, have reported a physiological relaxation effect of a forest compared with the effect of an urban environment, with findings such as increased parasympathetic nervous activity, decreased sympathetic nervous activity, and decreased cortisol levels and cerebral blood flow in the prefrontal cortex [8,9,10,11,12,13,14,15,16]. Moreover, residents who lived near a large amount of greenery were shown to have lower chronic stress levels than those who did not [17,18].

In laboratory experiments involving sensory stimuli, the relaxation effect of nature-derived stimulation has been reported. For example, studies have shown positive physiological effects when participants viewed images of a forest landscape [19], wooden materials [20], indoor plants [21,22,23,24], and flowers [25,26], which induced increased parasympathetic nervous activity, decreased sympathetic nervous activity, and decreased prefrontal cortex activity. Stress recovery is also faster in people who view natural scenes than in people who view urban scenes [27]. A number of studies have shown that viewing an image of nature through videos and virtual nature scenes results in decreased stress and increased positive emotions [28,29,30]. Relaxation has also been demonstrated with exposure to olfactory stimuli such as the scent of wood [31,32], fresh roses [33], and rose and orange essential oils [34]. The researchers demonstrated that these olfactory stimuli resulted in increases in parasympathetic nervous activity and decreases in prefrontal cortex activity. Tactile contact with wood resulted in similar physiological effects, increasing parasympathetic nervous activity and decreasing sympathetic nervous activity and prefrontal cortex activity [35,36,37].

These studies demonstrated that nature-derived visual, olfactory, and tactile stimuli affect the brain and autonomic nervous activities, with these changes inducing a state of relaxation in humans. However, there have been few studies on nature-derived auditory stimulation. One study showed that natural sounds tended to promote the recovery of skin conductance levels compared with road noise [38]. In another study on stress recovery effects, participants were shown a virtual reality image with or without natural sounds; viewing the image with natural sounds resulted in enhanced parasympathetic nervous activity and improved stress recovery [39]. As yet, no studies have investigated the effects of natural auditory stimuli on the indicators of brain and autonomic nervous activities. 

Recently, high-resolution sounds have been increasingly used in Japan. High-resolution sound sources are considered to provide highly realistic natural sounds. Although Oohashi et al. [40] have reported the effects of high-resolution sounds using electroencephalogram (EEG), the physiological effects of high-resolution sounds are rarely reported. This study aimed to compare the physiological effects of auditory stimulation with forest and city sounds using high-resolution sound sources.

## 2. Materials and Methods

### 2.1. Participants

The study recruited 29 female university students (mean age ± standard deviation, 22.3 ± 2.1 years) via a bulletin board at the university. The following exclusion criteria were applied: poor physical condition; a respiratory illness; menstruation on the day of the experiment; a hearing impairment; and smoking.

All participants were informed about the purpose and experimental procedures of the study and gave their informed consent. The study was conducted in line with the principles of the Declaration of Helsinki, and the protocol was approved by the Ethics Committees of the Center for Environment, Health and Field Sciences, Chiba University, Japan (project identification code number, 36). It was registered in the University Hospital Medical Information Network of Japan (UMIN ID: UMIN000034821).

### 2.2. Auditory Stimulation

To heighten realism, high-resolution sound sources were used as auditory stimuli. We selected the sound of a murmuring brook in the Togakushi forest in Nagano Prefecture as the forest auditory stimulus (forest sound). The other stimulus (city sound) was the sound of city traffic at the Shibuya intersection in Tokyo. The sounds were recorded using a high-resolution sound recorder, with a sample rate of 96 kHz and 24 bit quantization. 

The participants’ sensory evaluations of sound intensity were scored from 0 to 10: 0, inaudible sound; 2, faint sound; 4, quiet sound; 6, easy to hear sound; 8, loud sound; and 10, very loud sound. It was ensured that there was no significant difference in terms of the subjective sound intensity scores between the forest and city sounds (scores, 6.1 ± 0.1 and 6.0 ± 0.2, respectively). The auditory stimuli were played to the participants using headphones at 48.6 dB for the forest sound and 51.5 dB for the city sound.

### 2.3. Study Protocol

**Figure 1:**
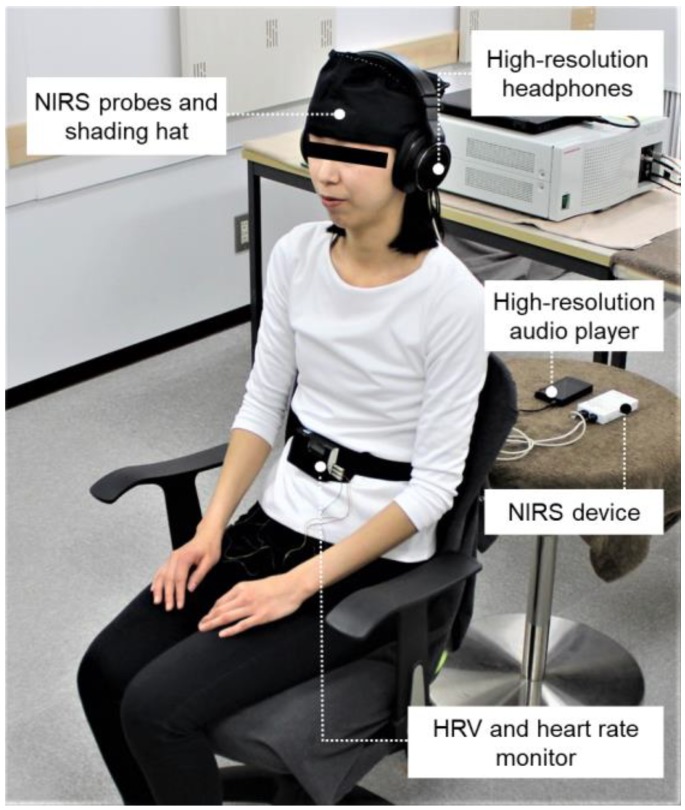
Experimental set-up for auditory physiological measurement. NIRS: near-infrared spectroscopy; HRV: heart rate variability.

**Figure 2:**
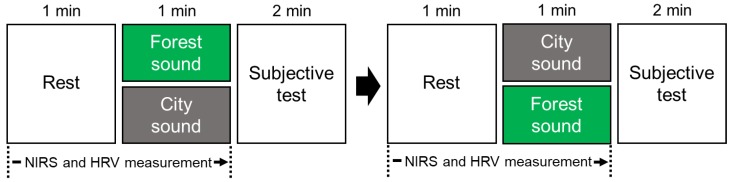
Measurement protocol. The order of the forest and city sounds was counterbalanced to avoid any order effects. NIRS: near-infrared spectroscopy; HRV: heart rate variability.

After receiving an explanation of the study details and protocol in the waiting room, each participant was moved into a chamber with an artificial climate for physiological measurement (maintained at 25 °C with 50% relative humidity and 200 lux illumination) at the Center for Environment, Health and Field Sciences, Chiba University. This ensured that the participants were exposed to minimal external influences and were tested under the same physical and soundproof conditions. Figure 1 shows the experimental set-up for the physiological measurement upon auditory stimulation, and Figure 2 summarizes the measurement protocol. The physiological measurement devices (heart rate variability (HRV), near-infrared spectroscopy (NIRS)) and headphones that provided auditory stimulation were attached to the participants. The participant was asked to rest, with eyes closed, for one minute, and then the auditory stimulus (forest or city sound) was provided for one minute. The forest and city sounds were provided in a counterbalanced order to eliminate any possible impact of the order on the physiological responses. The physiological activities were measured continuously during the rest and stimulation periods. After measuring the participants’ physiological responses, subjective tests were performed for about two minutes.

### 2.4. Physiological Measurements

#### 2.4.1. Near-Infrared Spectroscopy

When there is an increase in local brain activity, brain blood flow increases, resulting in luxury perfusion such that the brain blood flow exceeds oxygen consumption [41]. This produces a detectable increase in oxyhemoglobin (oxy-Hb) concentration. Changes in oxy-Hb concentrations are known to be consistent with the changes in blood flow in the brain [42], and it is thought that a decrease in oxy-Hb concentration is associated with physiological calming. In this study, near-infrared spectroscopy (NIRS) was used as an indicator of prefrontal cortex activity. Two NIRS probes (Pocket NIRS Duo, Dynasense, Shizuoka, Japan), attached to the left and right forehead, were used to detect changes in the concentrations of oxy-Hb and deoxygenated hemoglobin in the cerebral blood flow [43]. Concentrations in the left and right prefrontal cortex were recorded every second during the rest and auditory stimulation periods. Each data point was then expressed as the difference from the average of the 60 s rest period.

#### 2.4.2. Heart Rate Variability and Heart Rate

Heart rate variability (HRV) and heart rate were used as indicators of autonomic nervous activity [44,45]. Electrocardiography was performed using a portable electrocardiogram (Activtracer AC-301A; GMS, Tokyo, Japan), and HRV was analyzed for the periods between consecutive R waves (RR intervals) in the electrocardiogram. The power levels of the high-frequency (HF; 0.15–0.40 Hz) and low-frequency (LF; 0.04–0.15 Hz) components of HRV were calculated using the maximum entropy method (MemCalc/win; GMS, Tokyo, Japan) [46,47]. The HF component reflects parasympathetic nervous activity, and the LF/HF ratio reflects sympathetic nervous activity [44]. In this study, the natural logarithmic values of HF and the LF/HF power ratio (i.e., ln(HF) and ln(LF/HF), respectively) were used to normalize the participants’ HRV values [48]. The average HRV and heart rate values during the rest and stimulation periods were calculated.

Respiratory changes can influence HRV data; therefore, the participants’ respiratory rates were estimated during the period between the two stimuli. The respiratory rate can be estimated from the HRV power spectrum [49]. Generally, heart rate accelerates during inspiration and decelerates during expiration [50,51]; thus, the respiratory rate can be estimated from the dominant frequency of the HF component. We calculated the HRV power spectrum using the maximum entropy method and located the maximum power of the HF component using the associated frequency as the dominant respiratory frequency during the measurement period. To detect the peak frequency of the HF component, the model order for spectral analysis was chosen from the 7th to 12th orders, with the 9th order used in principle.

### 2.5. Psychological Measurements

The participants’ psychological feelings associated with each auditory stimulation were evaluated with two subjective tests using questionnaires. The first test used the semantic differential (SD) method [52]; the participant responded to three scales based on opposing adjective pairs (comfortable–uncomfortable, relaxed–aroused, and natural–artificial), each of which was evaluated on 13 scales. The Profile of Mood States (POMS) questionnaire was used to evaluate mood states from the scores for tension–anxiety (T-A), depression–dejection (D), anger–hostility (A-H), fatigue (F), confusion (C), and vigor (V) [53,54]. The total mood disturbance (TMD) score was calculated using this formula: (T-A) + (D) + (A-H) + (F) + (C) − (V) [54]. This score is practical from a clinical perspective, and is considered highly reliable because of the intercorrelations among the six primary POMS factors [53]. To reduce the burden on the participants, a shortened Japanese version of the POMS with 30 questions [55] was used.

### 2.6. Statistical Analysis

In the analyses of the physiological indices (NIRS, HRV, heart rate, and respiratory frequency), paired *t*-tests were used to compare the average values for the 60 s auditory stimulation periods between the forest and city sounds. The Wilcoxon signed-rank test was used in the analysis of the psychological measurements to compare the effects of the different stimuli. One-sided tests were used, based on the hypothesis that humans are relaxed by nature-derived auditory stimulation. The statistical package for the social sciences software (version 21.0, IBM, Armonk, NY, USA) was used, and *p-*values < 0.05 were considered statistically significant.

## 3. Results

### 3.1. Physiological Effects

#### 3.1.1. Near-Infrared Spectroscopy (NIRS)

**Figure 3:**
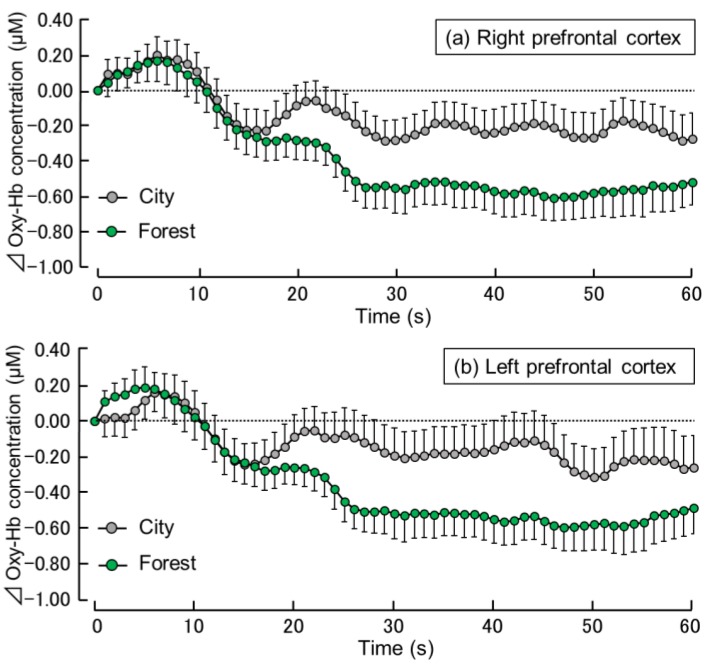
Time-dependent changes in the oxy-hemoglobin (oxy-Hb) concentrations in the right (**a**) and left (**b**) prefrontal cortex during 60 s exposure to the forest vs. city sounds. Data are expressed as mean ± standard error (n = 29).

Figure 3 shows the time-dependent changes in oxy-Hb concentration per second in the right and left prefrontal cortex during the exposure to the forest and city sounds. In the right prefrontal cortex (Figure 3a), changes in the oxy-Hb concentration were similar for the forest and city sounds for the first 15 s after exposure. From about 16 s, the oxy-Hb concentration was lower after the exposure to the forest sound compared with that after exposure to the city sound. From about 25 s to the end of the exposure period, the oxy-Hb concentration during the exposure to the forest sound remained at a fairly constant level, lower than that during the exposure to the city sound. Oxy-Hb concentrations in the left prefrontal cortex showed similar trends (Figure 3b).

**Figure 4:**
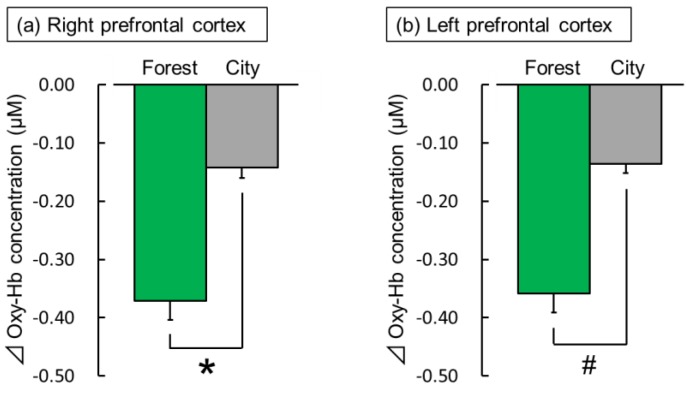
The overall mean oxyhemoglobin (oxy-Hb) concentrations in the right (**a**) and left (**b**) prefrontal cortex during exposure to the forest vs. city sounds. Data are expressed as mean ± standard error (n = 29). * *p* < 0.05, # *p* < 0.07 as determined by paired *t*-test (one sided).

Figure 4 shows the comparison of the mean oxy-Hb concentrations during the 60 s exposure to the forest and city sounds. Compared with the exposure to the city sound, the exposure to the forest sound significantly decreased oxy-Hb concentrations in the right prefrontal cortex (forest: −0.37 ± 0.03 µM (mean ± standard error); city: −0.14 ± 0.02 µM; t(28) = −2.36; *p* < 0.05; Figure 4a). In the left prefrontal cortex, a decrease was observed in the oxy-Hb concentration during the exposure to the forest sound compared to the exposure to the city sound (forest: −0.36 ± 0.03 µM; city: −0.14 ± 0.02 µM; t(28) = −1.59; *p* < 0.07; Figure 4b).

#### 3.1.2. Heart Rate Variability (HRV) and Heart Rate

**Figure 5:**
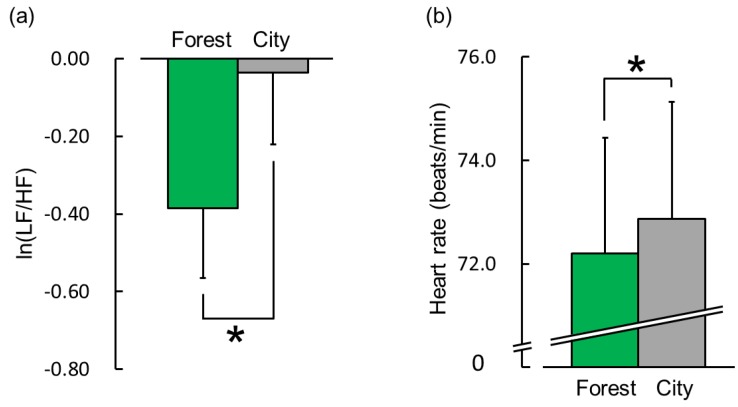
Indicators of sympathetic nervous activity and heart rate during exposure to the forest vs. city sounds for 60 s. (**a**) The overall mean natural log of the low-frequency to high-frequency power ratio of heart rate variability (ln(LF/HF)) and (**b**) overall mean heart rate. Data are expressed as mean ± standard error (n = 29). * *p* < 0.05 as determined by paired *t*-test (one sided).

The HRV data revealed a significant difference in terms of sympathetic nervous activity in response to exposure to the forest and city sounds. Figure 5a shows the overall mean ln(LF/HF) ratio during the 60 s exposure to the forest and city sounds. The ln(LF/HF) ratios were −0.38 ± 0.18 during the exposure to the forest sound and −0.04 ± 0.18 during the exposure to the city sound. This indicates that sympathetic nervous activity was significantly lower during exposure to the forest sound than that during exposure to the city sound (Figure 5a; t(28) = −2.39; *p* < 0.05). The mean baseline ln(LF/HF) ratios did not differ significantly between the forest (−0.08 ± 0.16) and city (−0.15 ± 0.21) sounds during the 60 s rest period (*p* > 0.05). The ln(LF) and ln(HF) were 5.28 ± 0.16 and 5.66 ± 0.17 in the forest sound and 5.53 ± 0.14 and 5.57 ± 0.18 in the city sound, respectively. Figure 5b shows the mean heart rate during the 60 s exposure to the two stimuli. The heart rate was 72.2 ± 2.2 bpm during exposure to the forest sound and 72.9 ± 2.3 bpm during exposure to the city sound, i.e., the heart rate was significantly lower during exposure to the forest sound than that during exposure to the city sound (Figure 5b; t(28) = −1.93; *p* < 0.05). The mean baseline heart rate did not differ significantly between the forest (73.1 ± 2.1 bpm) and city (73.4 ± 2.3 bpm) sounds during the 60 s rest period (*p* > 0.05). There was no significant difference in terms of the ln(HF) value between exposure to the two stimuli. Moreover, there was no significant difference in terms of respiratory frequency between the forest and city sounds (0.26 ± 0.01 Hz vs. 0.27 ± 0.01 Hz; t(28) = −1.76; *p* > 0.05). 

### 3.2. Psychological Effects

#### 3.2.1. Modified Semantic Differential (SD) Method

**Figure 6:**
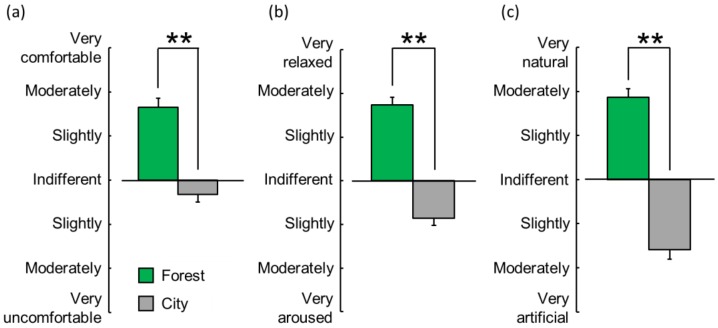
Subjective feelings on three scales (comfortable–uncomfortable (**a**), relaxed–aroused (**b**), and natural–artificial (**c**)), as measured by a modified semantic differential method, after exposure to the forest vs. city sounds. Data are expressed as mean ± standard error (n = 29). ** *p* < 0.01 determined by the Wilcoxon signed-rank test (one sided).

Figure 6 summarizes the results of the evaluation of the participants’ subjective feelings on the three scales, as measured by the modified SD method, after exposure to the forest vs. city sounds. For the comfortable–uncomfortable scale, the mean score was “slightly-to-moderately comfortable” when exposed to the forest sound but was “indifferent-to-slightly uncomfortable” when exposed to the city sound (Figure 6a, *p* < 0.01). Thus, the exposure to the forest sound induced a more comfortable feeling than exposure to the city sound. For the relaxed–aroused scale, the mean score was “slightly-to-moderately relaxed” with exposure to the forest sound but was “slightly aroused” with exposure to the city sound (Figure 6b, *p* < 0.01). Thus, the forest sound evoked a more relaxed feeling than the city sound. Further, for the natural–artificial scale, the mean score was “moderately natural” with exposure to the forest sound but was “slightly-to-moderately artificial” with exposure to the city sound (Figure 6c, *p* < 0.01). Thus, the forest sound evoked a more natural feeling than the city sound.

#### 3.2.2. Profile of Mood States (POMS)

**Figure 7:**
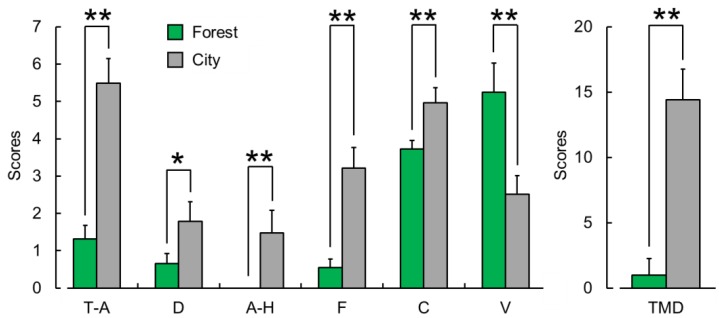
Subjective evaluation of mood states as measured by the six subscales and total mood disturbance score on the profile of mood states (POMS) questionnaire after exposure to the forest vs. city sounds. T-A, tension–anxiety; D, depression-dejection; A-H, anger–hostility; F, fatigue; C, confusion; V, vigor; and TMD, total mood disturbance. Data are expressed as mean ± standard error (n = 29). ** *p* < 0.01, * *p* < 0.05 as determined by the Wilcoxon signed-rank test (one sided).

Figure 7 summarizes the results of the POMS questionnaire, showing the mean scores for the six subscales and total mood disturbance score after exposure to the forest or city sounds. Scores for the negative subscales were significantly lower after exposure to the forest sound than those after exposure to the city sound (tension–anxiety (T-A, *p* < 0.01); depression–dejection (D, *p* < 0.05); anger–hostility (A-H, *p* < 0.01); fatigue (F, *p* < 0.01); and confusion (C, *p* < 0.01)), whereas the positive mood state of vigor (V, *p* < 0.01) was significantly higher. The total mood disturbance (TMD) score was significantly lower after exposure to the forest sound than that after exposure to the city sound (*p* < 0.01).

## 4. Discussion

This study examined the physiological effects of exposure to forest sound on brain and autonomic nervous activities by measuring oxy-Hb concentration in the prefrontal cortex and HRV. Psychological effects were examined through subjective assessments using the modified SD method and POMS questionnaire. Compared with exposure to the city sound, exposure to the forest sound resulted in decreased oxy-Hb concentrations in the right prefrontal cortex and suppression of sympathetic nervous activity and heart rate. Psychologically, exposure to the forest sound resulted in greater feelings of comfort, relaxation, and naturalness, as well as improved mood states, than exposure to the city sound. Taken together, these physiological and psychological responses indicate that nature-derived auditory stimulation induced relaxation effects.

The finding of decreased right prefrontal cortex activity with the exposure to the forest sound was consistent with the results of previous studies on the physiological responses to visual, olfactory, and tactile stimuli [19,26,34]. These studies reported decreased oxy-Hb concentrations in the right prefrontal cortex when viewing a forest image (vs. a city scene) [19], viewing fresh roses (vs. no stimulus) [26], and smelling rose and orange essential oils (vs. odorless air) [34]. The present study’s findings provide further confirmation about the calming effect of exposure to a nature-derived auditory stimulation on the prefrontal cortex activity.

The HRV results showed a significant decrease in sympathetic nervous activity during exposure to the forest sound. This was consistent with the results of previous studies that investigated the physiological visual effects of exposure to nature [21,23,24,56,57]. These studies reported decreases in sympathetic nervous activity when viewing real pansies (vs. artificial pansies) [56], bonsai (vs. no stimulus) [21,23], foliage plants (vs. no stimulus) [24], and a three-dimensional image of a water lily (vs. a two-dimensional image) [57]. However, the present study found no significant difference in terms of parasympathetic nervous activity during exposure to the forest and city sounds. In contrast, some previous studies have shown significant enhancement in parasympathetic nervous activity with nature-derived stimuli [21,25,32,33,35,36,37]. The reason for the discrepancy between the sympathetic and parasympathetic nervous activities in the present study remains unknown, and this issue requires further study. The present study also found a decrease in heart rate with exposure to the forest sound compared with that to the city sound. This is consistent with the findings of previous studies on the physiological effects of nature-derived stimuli [32,37]. A similar finding was reported in a recent clinical study on patients hospitalized in a cardiac care unit; in the patient group that listened to natural sounds for 30 min, there were significant decreases in heart rate before and after the intervention [58].

A log-transformed LF to HF ratio (ln [LF/HF]) significantly decreased during exposure to forest sound. In this study, LF/HF was considered as an indicator of the relative sympathetic nervous activity. Hence, the present results imply that exposure to forest sound decreases the relative sympathetic nervous activity. This supposition is supported by a significantly lower mean HR during exposure to forest sound. However, this issue remains controversial among researchers. In recent years, some researchers have suggested that the LF of HRV is the result of modulation by baroreflex rather than an index of sympathetic activity [59,60]. On the other hand, this idea has been refuted by other researchers [61]. Considering that this study is not about the physiological interpretation of HRV, we did not specifically discuss this point. The uncertainty of the physiological interpretation of LF/HF is one of the limitations of this study. 

The study focused on shorter HR recordings. For short-term HRV measurement, 5 min recording is generally recommended [44]. To investigate acute physiological responses to sound stimulation, we analyzed the HR recording every 60 s. The HRV indicator became unstable due to the short HR recording. The instability of HRV indices in this study increased the probability of Type II errors (false negatives) in statistical tests, but did not increase the probability of Type I errors (false positives). Therefore, the short duration of the HRV measurements did not impair the reliability of the present results that demonstrated a significant decrease in ln(LF/HF) during exposure to forest sounds.

The results of the psychological assessments using the modified SD method showed that exposure to the forest sound increased psychological relaxation, eliciting greater feelings of comfort, relaxation, and naturalness compared with exposure to the city sound. These results are similar to those of previous studies that have compared the effects of forest and city stimuli in field and laboratory experiments [8,9,10,11,12,13,14,15,16,19]. The POMS questionnaire scores of mood states showed that forest sounds can relieve psychological tension, depression, anger, fatigue, and confusion compared with city sounds, and that they could enhance psychological vigor. Previous studies that investigated the psychological effects of exposure to nature-derived stimuli via other senses have reported similar improvements in mood states [20,22,25,26,36,62]. Several previous studies on music therapy reported its positive effect on mood states, such as in reducing anxiety levels in surgical patients [63] or facilitating a positive change in calm–anxious, energetic–tired, and agreeable–hostile mood states of adults with neurological impairments [64]. The present study demonstrated that the forest sound provided psychological benefits of positive feelings and mood state improvement similar to the effects of music therapy.

In summary, this study showed both physiological and psychological relaxation associated with exposure to the forest sound compared with exposure to the city sound. However, the present study has certain limitations that should be considered. First, we examined the effects of nature-derived auditory stimulation only in women in their 20s. To generalize these effects, further studies are needed with other demographic groups, including individuals of different ages and men. Second, although various natural sounds, such as the sound of the wind, a brook, murmuring trees, or singing birds [65], are associated with forests, we used the sound of a brook as the auditory stimulus. Further studies that use other kinds of natural sounds are needed. 

## 5. Conclusions

The findings of the present study indicated that, when compared with city sounds, exposure to the forest sound induced physiological and psychological relaxation, with decreased oxy-Hb concentrations in the right prefrontal cortex activity, decreased sympathetic nervous activity, lower heart rate, and increased feelings of comfort, relaxation, and naturalness, as well as improved mood states. The study clarified that a nature-derived auditory stimulation induced physiological and psychological aspects of relaxation compared with city-derived auditory stimulation.
